# 58 Objective and Subjective Melanin Measurements Within Dyschromic Scars Do Not Correlate: A Treatment Evaluation Problem

**DOI:** 10.1093/jbcr/irae036.050

**Published:** 2024-04-17

**Authors:** Monica Collins, Jennifer Huang, Taryn E Travis, Melissa M McLawhorn, Jeffrey W Shupp, Bonnie C Carney

**Affiliations:** Georgetown University School of Medicine, Washington, District of Columbia; MedStar Washington Hospital Center, Georgetown University School of Medicine, Washington, DC; MedStar Health Research Institute, Washington, District of Columbia; MedStar Health |Georgetown University School of Medicine, Washington, DC, DC; Georgetown University School of Medicine, Washington, District of Columbia; MedStar Washington Hospital Center, Georgetown University School of Medicine, Washington, DC; MedStar Health Research Institute, Washington, District of Columbia; MedStar Health |Georgetown University School of Medicine, Washington, DC, DC; Georgetown University School of Medicine, Washington, District of Columbia; MedStar Washington Hospital Center, Georgetown University School of Medicine, Washington, DC; MedStar Health Research Institute, Washington, District of Columbia; MedStar Health |Georgetown University School of Medicine, Washington, DC, DC; Georgetown University School of Medicine, Washington, District of Columbia; MedStar Washington Hospital Center, Georgetown University School of Medicine, Washington, DC; MedStar Health Research Institute, Washington, District of Columbia; MedStar Health |Georgetown University School of Medicine, Washington, DC, DC; Georgetown University School of Medicine, Washington, District of Columbia; MedStar Washington Hospital Center, Georgetown University School of Medicine, Washington, DC; MedStar Health Research Institute, Washington, District of Columbia; MedStar Health |Georgetown University School of Medicine, Washington, DC, DC; Georgetown University School of Medicine, Washington, District of Columbia; MedStar Washington Hospital Center, Georgetown University School of Medicine, Washington, DC; MedStar Health Research Institute, Washington, District of Columbia; MedStar Health |Georgetown University School of Medicine, Washington, DC, DC

## Abstract

**Introduction:**

Dyschromia, the presence of hyper- and/or hypopigmented epidermis, is a common result of burn injury and is associated with negative aesthetic outcomes. Three clinical scales, the Patient and Observer Scar Assessment Scale - Patient (POSAS-P), Patient and Observer Scar Assessment Scale - Observer (POSAS-O), and modified Vancouver Scar Scale (mVSS) are used to evaluate dyschromia when assessing treatment options and outcomes. However, these assessment tools have yet to be validated against objective scar tissue melanin measurements. We hypothesize that these scar scales do not adequately capture pigmentation changes, necessitating more accurate metrics for clinical assessment.

**Methods:**

Patients (n=98) with dyschromic burn scars due to thermal, chemical, electrical, or friction burns were recruited from a local burn center. Scars were rated on the POSAS-P, POSAS-O, and mVSS and the melanin content of uninjured skin and hyper-pigmented scar tissue was assessed using a Skin Color Catch non-invasive probe. The “degree of hyperpigmentation” (DOH) was calculated by the change in melanin content from the normal skin (NS) to the hyper-pigmented scar tissue, which was tested for correlation with POSAS-P-color and POSAS-O-pigmentation scores. The DOH between scars in each of the four categories of the mVSS-pigment was compared. For POSAS-P and POSAS-O, a higher score indicates more dyschromia. For mVSS, dyschromia is categorized.

**Results:**

Hyperpigmented scars were on average 96.08±6.08 Melanin Index points higher than NS. Observers rated hyperpigmented scars high at 7.14±0.28 while patients rated higher at 7.67±0.24 (both out of 10). The most common mVSS-pigment category observed was 2, mixed pigmentation. The correlations between POSAS-P-color and DOH and POSAS-O-pigmentation and DOH were not significant (r=-0.033, p=0.7455 and r=-0.12, p=0.2323). Additionally, there were no significant differences in melanin index means between scars in the four mVSS-pigment categories (p < 0.05).

**Conclusions:**

The non-significant correlation between the scoring value from the POSAS-P, POSAS-O, and mVSS with DOH indicate that these tools do not adequately capture degree of dyschromia in patients with dyspigmentation following burn injury.

**Applicability of Research to Practice:**

The data from these scales should not solely be used to evaluate treatment effect.

